# *Staphylococcus aureus* intramammary challenge in non-lactating mammary glands stimulated to rapidly grow and develop with estradiol and progesterone

**DOI:** 10.1186/s13567-018-0542-x

**Published:** 2018-06-05

**Authors:** Benjamin D. Enger, Carly E. Crutchfield, Taylor T. Yohe, Kellie M. Enger, Stephen C. Nickerson, Catherine L. M. Parsons, Robert Michael Akers

**Affiliations:** 1 Dairy Science Department, Virginia Polytechnic Institute and State University, Blacksburg, VA 24060 USA; 2 Animal and Dairy Science Department, University of Georgia, Athens, GA 30602 USA

## Abstract

Intramammary infections (IMI) are prevalent in non-lactating dairy cattle and their occurrence during periods of significant mammary growth and development (i.e. pregnant heifers and dry cows) is believed to interfere with growth, development, and subsequent milk production. However, direct study of IMI impacts on non-lactating but developing mammary glands is lacking. The objectives of this study were to (1) define how IMI affected total and differential mammary secretion somatic cell counts in mammary glands stimulated to rapidly grow using estradiol and progesterone, and (2) characterize changes in mammary morphology in response to IMI. Mammary growth was stimulated in 19 non-pregnant, non-lactating cows and 2 quarters of each cow were subsequently infused with either saline (*n* = 19) or *Staphylococcus aureus* (*n* = 19). Mammary secretions were taken daily until mammary tissues were collected at either 5 or 10 days post-challenge. *Staph. aureus* quarter secretions yielded greater concentrations of somatic cells than saline quarters and contained a greater proportion of neutrophils. *Staph. aureus* mammary tissues exhibited higher degrees of immune cell infiltration in luminal and intralobular stroma compartments than saline quarters. Infected tissues also contained reduced areas of epithelium and tended to have greater amounts of intralobular stroma. Results indicate that IMI in non-lactating glands that were stimulated to grow, produced immune cell infiltration into mammary tissues and secretions, which was associated with changes in mammary tissue structure. The observed reduction of mammary epithelium indicates that IMI impair mammary development in rapidly growing mammary glands, which may reduce future reduced milk yields.

## Introduction

Bovine mastitis is almost exclusively the result of a bacterial intramammary infection (IMI) and continues to be a major challenge for the US and global dairy industries. The effects of mastitis are most apparent, and appreciated, in lactating cattle given the considerable volume of literature that has described the increase in milk somatic cell count (SCC) [1], reduced milk yields [2], and histopathological changes [3] that occur in response to IMI. Regardless of these well-documented effects in the lactating bovine mammary gland, it is recognized that both non-lactating heifers [4, 5] and dry cows [6] can also develop IMI.

Quarter IMI prevalence in nulliparous heifers is estimated to be approximately 43%, based on a weighted average of summarized survey studies [7] and approximately 8–25% of quarters in dry cows are expected to acquire new infections during the dry period between lactations [8]. The occurrence of such IMI in non-lactating mammary glands is concerning given the considerable mammary growth that occurs during these distinct physiological states. For instance, the greatest amount of mammary growth and development that transpires during an animal’s life occurs during first gestation in preparation for lactation [9]. Significant growth also occurs in dry cow mammary glands between lactations, contributing to the observed increased milk yields in successive lactations [10, 11]. Previously, the effects of IMI in non-lactating, non-pregnant, mammary growth quiescent heifer glands were examined, and marked changes in glandular structure such as reduced areas of epithelium and increased mammary stroma were described [12]. These results demonstrated that IMI negatively affects non-lactating mammary gland structure, which is expected to impact mammary function (future growth and development and milk yields). Despite this recognition that IMI impacts non-lactating mammary glands, no studies have investigated how IMI influences histological or morphometric changes in glands that are rapidly growing and developing.

The mammary growth and development occurring during first gestation and subsequent dry periods is largely attributed to the key pregnancy associated hormones, estradiol and progesterone. Substantial literature describes the pivotal roles that these hormones have in driving mammary epithelial cell proliferation [13] and glandular morphogenesis [14–16] to support subsequent lactation and have been reviewed previously [17]. Given the key role of these hormones, their utility, in a model setting, to stimulate rapid mammary growth and development so that molecular mechanisms involved in mammary growth and development may be elucidated shows promise.

It is logical to suspect that an IMI occurring when mammary parenchyma is rapidly growing and developing, (i.e., in the heifer during late gestation or in the multiparous cow during the second half of the dry period) would be problematic. In short, a key question remains: Is rapid mammogenesis compatible with the immune responses initiated to combat a newly developed IMI, and can the processes of mammogenesis and IMI eradication coexist without consequence? If there are conflicts, how are these manifested? While there are differences in the dynamics between the mammary growth experienced in late gestation heifers (parenchyma expansion into the fat pad) and dry cows during the dry period (cessation of milk secretion, regression, and redevelopment, etc.), both situations entail rapid mammogenesis, driven by estradiol and progesterone, to establish the parenchymal tissue necessary for lactation. In this study, non-pregnant, dry cows, that had undergone a more extensive involution than that typically experienced by a dry cow due to the lack of concurrent pregnancy [18], were injected with estradiol and progesterone to stimulate rapid mammogenesis. Subsequently, animals were challenged with *Staphylococcus aureus* (*Staph. aureus*) to characterize how presence of IMI influences mammary morphology. The specific objectives of this study were to quantify total and differential somatic cells in mammary secretions from saline and *Staph. aureus* infused mammary glands and define the infiltration of immune cells into mammary tissues. Additionally, a histological evaluation was applied to characterize mammary tissue structure to determine if IMI influenced mammary development.

## Materials and methods

### Animal selection and study design

This work was approved by the Virginia Polytechnic Institute and State University Institutional Animal Care and Use Committee (Protocol #15-196). A total of 19 animals were selected from the Virginia Tech milking dairy herd for this study, and included non-pregnant, clinically healthy Holstein or Jersey cows that were being culled for reproductive or production reasons, not for reasons concerning udder health. Selected cows were identified from a larger cohort of milking cows for study inclusion by collecting 3 aseptic quarter foremilk samples, with 1 day between each sampling [19] during the last week of lactation for bacterial examination and SCC quantification. Bacterial examination followed the methods outlined by the National Mastitis Council [20] in which a 10-µL aliquot of fresh milk was streaked onto blood agar plates (Columbia Blood Agar, Hardy Diagnostics, Santa Maria, CA, USA). A local Dairy Herd Information Association Laboratory quantified milk somatic cells using a Fossomatic™ FC (FOSS North America, Eden Prairie, MN, USA). Quarters were classified as infected if 2 of the 3 samples taken were culture positive for the same pathogen [21].

To be included in the study, cows must have had at least 2 uninfected quarters. Furthermore, cows producing foremilk SCC ≤ 200 000/mL for all quarter samplings were preferentially selected over cows yielding higher SCC; none of the utilized animals were treated for *Staph. aureus* IMI during the current lactation. Cows were identified and dried off in groups due to limited animal availability. The first group, Group A, contained 6 cows; Groups B and C contained 9 and 4 cows, respectively (Table 1). All quarters of all cows were aseptically infused via the partial insertion technique [22] with a commercial dry cow therapy product (ToMORROW^®^, Boehringer Ingelheim Vetmedica Inc., St. Joseph, MO, USA) at dry-off and then moved to pasture. At 35 days dry, cows were administered a single dose of dinoprost tromethamine (Zoetis, Parsippany, NJ, USA) to synchronize animals to a similar day of estrus and relocated to a sawdust bedded barn. All quarters were aseptically sampled again at 39, 41, and 43 days dry for bacterial examination to confirm that at least 2 quarters were free of IMI.

**Table 1:** Experimental animal group demographics and corresponding ***Staph. aureus*** Novel inoculums infused into challenge quarters

Group	Breed	Mean last week milk SCC (cells/mL)	Lactations completed	Mean days in milk	Mean last week milk yield (kg/day)	*Staph. aureus* inoculum (CFU)
Group A	Holstein (*n* = 6)	141 000	1 (*n* = 5)	568	25.7	4300
			3 (*n* = 1)			
Group B	Holstein (*n* = 6); Jersey (*n* = 3)	126 000	1 (*n* = 7)	391	28.4	6400
			2 (*n* = 1)			
			6 (*n* = 1)			
Group C	Holstein (*n* = 4)	251 000	1 (*n* = 2)	611	22.0	7500
			2 (*n* = 1)			
			4 (*n* = 1)			

Cows began a mammary growth induction protocol at 45 days after dry off. The first day of this induction protocol marks the beginning of the core experimental approach used in this trial and will hereafter be referred to as day 1. Cows received consecutive daily injections of estradiol and progesterone on days 1 through 7 as described below to stimulate mammary growth and development. Cows were aseptically sampled again on days 8 and 9 to confirm that mammary glands remained culture negative. Two culture negative quarters from each cow were randomly assigned to receive either an intramammary infusion of sterile phosphate buffered saline (PBS) (*n* = 19) or *Staph. aureus* (*n* = 19). Secretion samples were taken immediately before intramammary infusion on day 10, and then again on days 11, 12, 13, 14, 16, 18, and 20 for bacterial examination and quantification and differentiation of somatic cells. During this sampling period, cows were randomly selected for euthanasia at either 5 days post-challenge (day 15; *n* = 10) or 10 days post-challenge (day 20; *n* = 9) for collection of mammary tissues.

### Estradiol and progesterone injections

Cows were administered daily subcutaneous injections of estradiol (0.1 mg/kg BW; Sigma-Aldrich Co., St. Louis, MO, USA) and progesterone (0.25 mg/kg BW; Sigma-Aldrich Co.) on alternating sides of the neck on days 1 through 7 [23]. Estradiol and progesterone were dissolved in absolute ethanol, mixed with benzyl benzoate, and sterilized using a 0.45-µm filter. The filtrate was then mixed with autoclaved corn oil, which served as the main injectable carrier. The final injectable solution, containing dissolved estradiol and progesterone, was 10% ethanol, 20% benzyl benzoate, and 70% corn oil by volume.

### Mammary secretion sampling and examination

Mammary secretion samples were obtained from cows by removing gross debris from teats and the base of the udder via a single-use paper towel. Teats were dipped in a commercial iodine teat disinfectant (TEAT-KOTE 10/III, GEA United States, Colombia, MD, USA), which remained on teat skin for at least 30 s [24] before removal with a single-use paper towel. Teat ends were scrubbed with 10 × 10 cm^2^ cotton squares soaked in 70% ethanol, and mammary secretions were aseptically expressed into sterile 5-mL round bottom polystyrene tubes. Secretions were immediately placed on ice and transported to the laboratory for culture and somatic cell quantification and differentiation.

Secretion samples were first processed for bacteriological examination using the methods discussed earlier and then used to determine somatic cell concentration (cell/mL) and then differentiate somatic cells. Somatic cells were quantified using the methods outlined by the National Mastitis Council Subcommittee on Screening Tests [25]. Fresh secretion samples were first diluted either 1:4 (Group A) or 1:10 (Group B and C) in PBS containing 2.2% bovine serum albumin (BSA). Duplicate smears were prepared by spreading 10 µL of the diluted secretion within a 1-cm^2^ circle on milk somatic cell counting slides (Bellco Glass Inc., Vineland, NJ, USA) and then dried at 45 °C on a slide warmer. Smears were subsequently stained for 2 min by flooding the slide with Newman’s Modified Stain Solution (Sigma-Aldrich Co.). Slides were drained of excess stain, dried, and rinsed in 3 changes of tap water. Stained smears were visualized under oil immersion with a 5-mm square reticle (Microscope World, Carlsbad, CA, USA), which produced a countable strip width of 0.050 mm for the microscope and reticle combination. Stained cells were enumerated by counting the number of cells across the diameter of the circle (11.28 mm) within the defined strip width. Each smear was counted by 2 independent counters; thus, a total of 4 counts were completed per secretion sample. Enumerated smears were used to calculate the SCC of the undiluted secretion sample, and these SCC were averaged to produce a single SCC estimate for each secretion sample. Final secretion SCC were log_10_ transformed.

The procedures used to differentiate secretion somatic cells were adapted from those described previously [26]. Briefly, 10 µL of fresh secretion was loaded into the top and bottom chambers of a double cytocentrifuge funnel, and 70-µL of PBS containing 2.2% BSA was added to each chamber. The cytocentrifuge funnel, fitted with a slide, was centrifuged at 110 × *g* in a Shandon CytoSpin 2 (Thermo Fisher Scientific, Waltham, MA, USA) for 10 min, which produced duplicate smears of the same sample on each slide. Slides were dried at room temperature then stained with a Wright–Giemsa stain (Electron Microscopy Sciences, Hatfield, PA, USA) for 2.5 min. Slides were then drained of excess stain, placed in a stain primed phosphate buffer (6.8 pH; electron microscopy sciences) for 4 min, rinsed with deionized water, and dried again at room temperature. Stained slides were coverslipped and somatic cells were visualized and differentiated by a single operator and classified as being either: (1) neutrophils; (2) macrophages, which would have included any epithelial cells present; or (3) lymphocytes. A total of 100 cells were differentiated for each duplicate smear resulting in 200 cells being differentiated and used to calculate percentages for each cell type.

### Intramammary challenge

The *Staph. aureus* Novel strain [27] was used as the challenge organism because of its demonstrated ability to induce apoptosis of bovine mammary epithelial cells in vitro [28]. Briefly, a single colony of *Staph. aureus* Novel was removed from a Columbia Blood Agar plate that had been incubated for 24 h and placed in a flask containing trypticase soy broth (Becton, Dickinson and Company, Franklin Lakes, NJ, USA). The inoculated flask was incubated at 37 °C for 6 h in a shaking incubator rotating at 250 rpm [29]. At the end of the incubation, the bacterial culture was centrifuged at 1600 × *g* for 10 min at 4 °C. The resulting pellet was resuspended in sterile PBS and washed twice more using these same procedures. The final bacterial pellet was resuspended in sterile PBS to a concentration of 5 × 10^8^ colony forming units (CFU)/mL based on absorbance measured at 600 nm. The adjusted *Staph. aureus* Novel suspension was serially diluted with sterile PBS to a target concentration of 5000 CFU/mL. For intramammary infusion, 1 mL of the final dilution was aseptically loaded into tuberculin syringes and transported to the farm on ice. The actual number of *Staph. aureus* Novel CFU infused into mammary glands was confirmed by diluting and plating the final dilution on trypticase soy agar (Becton, Dickinson and Company) and is reported for each group of cows in Table 1.

In preparation for infusion, gross debris was removed from teats and the base of the udder via a disposable paper towel, and teats were disinfected using a commercial aerosol teat disinfectant (Fight Bac, Deep Valley Farm Inc., Brooklyn, CT, USA). Teats were dried using a single use paper towel after allowing a teat disinfectant contact time of at least 30 s, and teats ends were then scrubbed with 10 × 10 cm^2^ cotton squares soaked in 70% ethanol. Aseptic secretion samples were collected and teats were cleaned again using the commercial aerosol teat disinfectant and cotton squares used before infusion. All quarters were infused via the partial insertion method using sterile teat cannulas (Jorgenson Labs, Loveland, CO, USA) affixed to loaded syringes. Quarters assigned to the saline treatment were always infused before *Staph. aureus* challenge quarters. Gloves were changed between cows and if gloves became soiled.

### Tissue collection and processing

Cows were euthanized by captive bolt and exsanguination for tissue collection. Udders were labeled for orientation, removed, cleaned of gross debris using paper towels, and placed on aluminum dissecting trays with the teats facing upward for dissection. Mammary parenchyma tissues were collected from saline and *Staph. aureus* infused quarters. Mammary tissues were collected from parenchyma proximal to the teat, dorsal to the gland cistern for histological evaluation and fixed in 10% formalin for 72 h. Formalin fixed tissues were transferred and stored in 70% ethanol before being dehydrated in a graded ethanol series and embedded in paraffin using an automated tissue processor (Leica TP 1020; Leica Biosystems Inc, Buffalo Grove, IL, USA).

### Tissue histologic analysis

Paraffin embedded mammary tissues were sectioned 5 µm thick using a rotary microtome (Model HM 340 E, Microm International GmbH, Waldoff, Germany) and floated in a water bath at 42 °C. Relaxed sections were mounted on Superfrost™ Plus microscope slides (Thermo Fisher Scientific), drained, and dried at 37 °C for 24 h on a slide warmer. Slides were deparaffinized in 3 changes of a xylene substitute (Clear-Rite™ 3, Thermo Fisher Scientific) for 5 min each and rehydrated to deionized water using a graded ethanol series. Sections were subsequently stained with hematoxylin and eosin and coverslipped using the procedures described previously [30].

**Figure 1:**
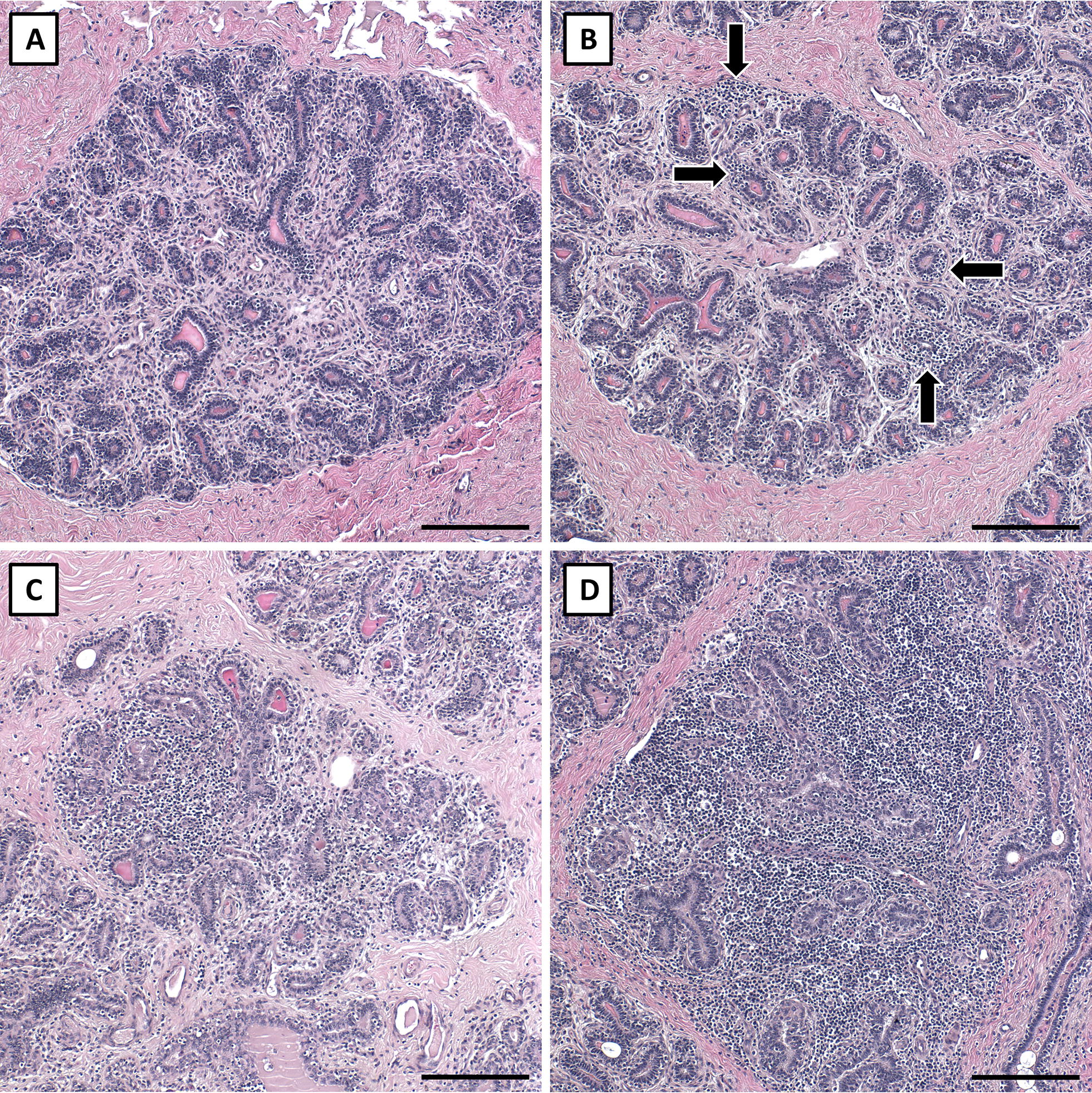
**Intralobular stromal immune cell invasion scoring.** The presented quarter lobules were used to characterize the degree of intralobular stromal immune cell invasion observed in saline and *Staph. aureus* quarter lobules; scores 1–4. **A** Depicts a score of 1 with no infiltration; **B** depicts a score of 2 where small isolated pockets of infiltration (arrows) are present; **C** exemplifies a score of 3 where cellular infiltration affects approximately 1/3 of the lobule; and **D** depicts a score of 4, marked infiltration affecting more than 2/3 of the lobule. Scale bars = 200 µm.

**Figure 2:**
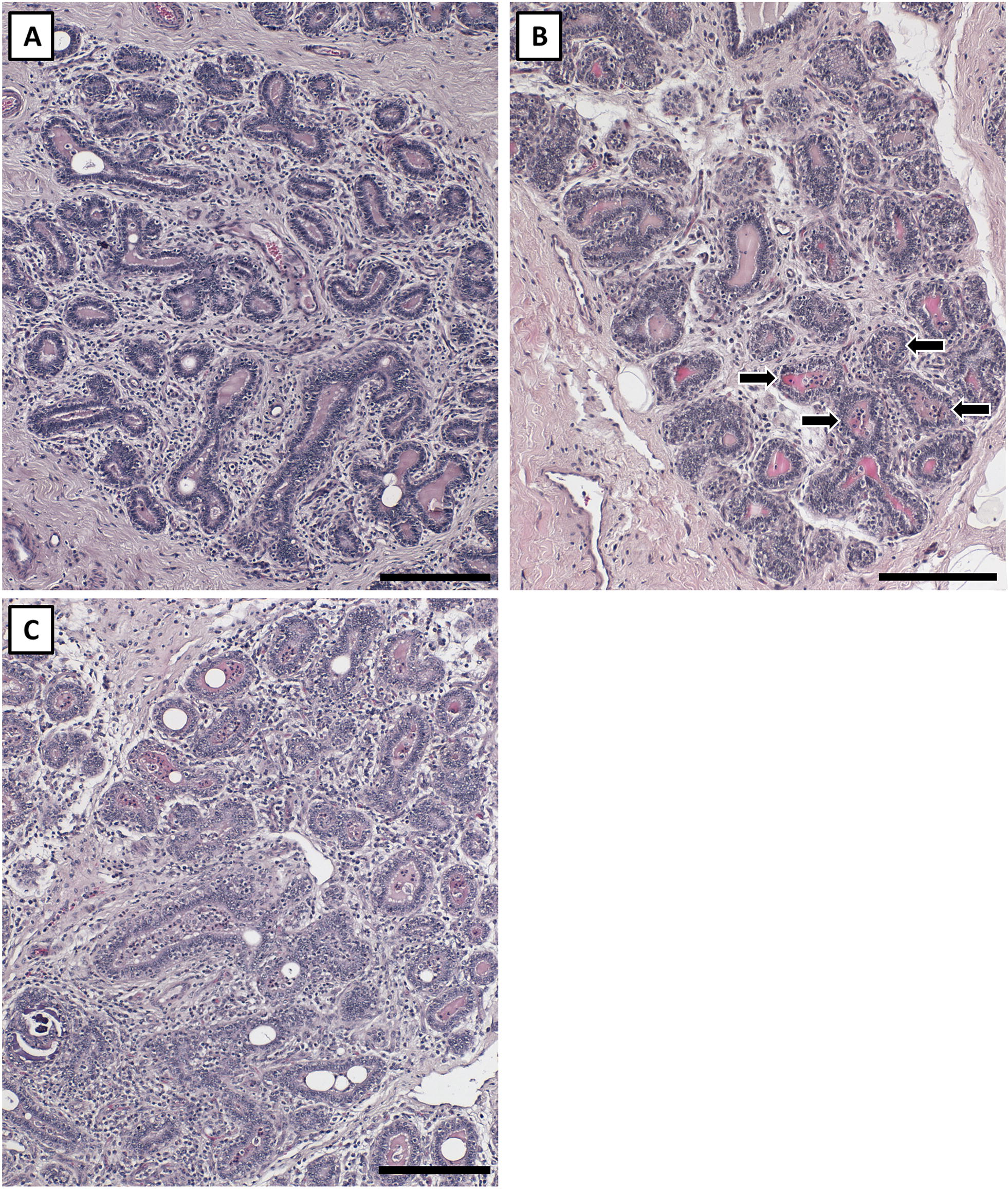
**Luminal immune cell invasion scoring.** The presented quarter lobules were used to characterize the degree of luminal immune cell invasion observed in saline and *Staph. aureus* quarter lobules; scores 1–3 are presented. **A** Depicts a score of 1 which signifies no luminal infiltration; **B** depicts a score of 2 with infiltration in fewer than half the lobule lumens (arrows); and **C** denotes a score of 3 which is marked infiltration in most lumens. Scale bars = 200 µm.

A single hematoxylin and eosin stained section for each experimental quarter was visualized in its entirety, and 8 representative lobules were identified and imaged at 100× to capture a representative profile of the lobules in each section. Areas of interlobular stroma were avoided, and only lobules were imaged; this was done to focus this analysis on the functional epithelium within the mammary gland. Imaged lobules were classified by a scorer blinded to treatments using a graded scoring scale to characterize the degree of immune cell infiltration in intralobular stroma and luminal area compartments independently. Intralobular stroma infiltration scores ranged from 1 to 4; a score of 1 was a lobule with no evidence of immune cell infiltration (Figure 1, score 1) and a score of 4 was a lobule that was more than 2/3 invaded by immune cells (Figure 1, score 4). Luminal infiltration scores ranged from 1 to 3; a score of 1 was a lobule that had scant infiltration of immune cells into luminal spaces (Figure 2, score 1) and a score of 3 reflected a lobule exhibiting marked luminal infiltration where almost all lumens contained immune cells (Figure 2, score 3).

**Figure 3:**
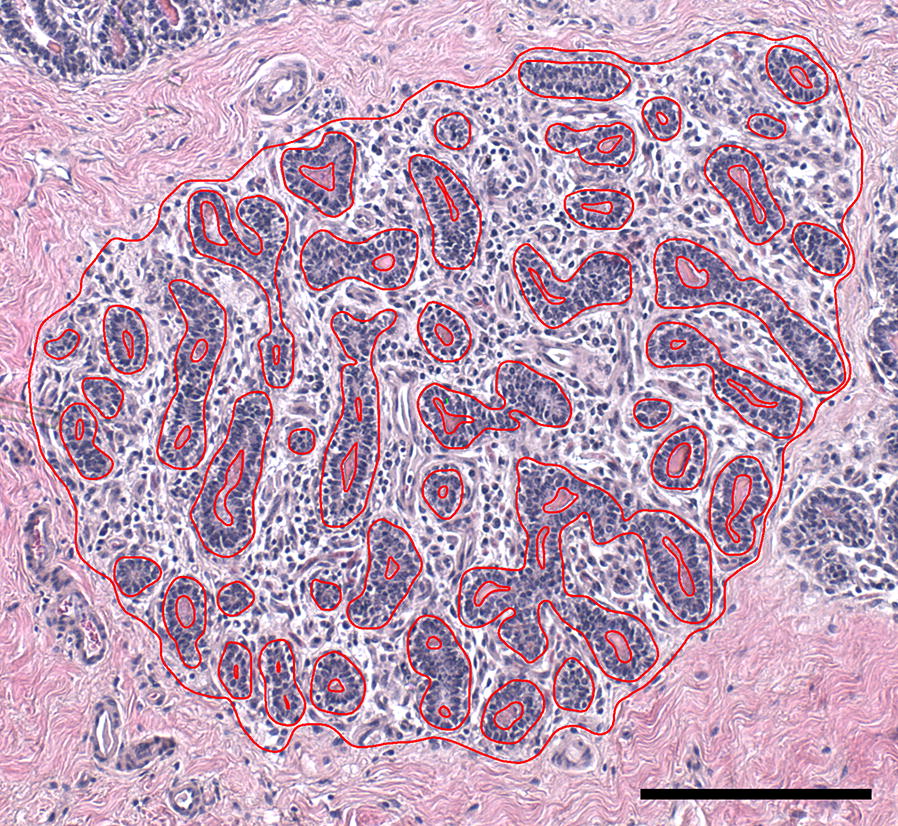
**Measurement of lobule tissue area percentages in imaged lobules.** Example image of the tracings applied to imaged lobules to measure lobule, intralobular stroma, epithelial, and luminal areas. Scale bar = 200 µm.

A second examination of these same imaged lobules was conducted to measure the area occupied by different tissue structures, e.g., epithelium, intralobular stroma, and lumen. Lobule and intralobular tissue structures were traced and measured using Image-Pro Plus 7.0 (Media Cybernetics, Rockville, MD, USA; Figure 3). This was achieved by first tracing and measuring the area of an imaged lobule and subsequently tracing and measuring the epithelial tissue structures contained within the lobule as they interface with the intralobular stroma. Therefore, these epithelial tracings would include the epithelial structure itself and any luminal areas contained within the structure. Finally, lumens alone, within the original traced lobule and epithelial structures, were traced, measured, and recorded. These tissue areas were used to calculate the epithelial area alone and the intralobular stromal area for each lobule.

### Statistical analysis

Total secretion SCC were analyzed using the MIXED procedure in SAS 9.4 (SAS Institute Inc., Cary, NC, USA) using log_10_ transformed SCC as the dependent variable. The final model included the fixed, independent effects of quarter treatment (*n* = 2), trial day when secretion was collected (*n* = 8), and their interaction. Cow nested within day of euthanasia and cow nested within day of euthanasia interacting with quarter treatment were included as random effects. Group of cows (*n* = 3) was not included as a random effect because it was non-significant when tested as a fixed effect (*P* > 0.05). Total log_10_ SCC were analyzed using a repeated measures approach where trial day served as the repeated time point and cow nested within day of euthanasia interacting with quarter treatment was defined as the measure repeated. Least squares means estimated by the model were compared using a slice procedure to determine if differences existed between treatments within the days secretions were collected.

Differential cell counts were also analyzed using the MIXED procedure of SAS using either, neutrophil, macrophage, or lymphocyte cell type percentages as the dependent variable. Each cell type was analyzed in a separate model. The models used to analyze the respective cell types were identical to the model used to analyze the total secretion SCC. A slice procedure was again used to compare the estimated least squares means for each treatment within day of secretion collection.

Immune cell intralobular stromal invasion and luminal invasion scores were averaged across the 8 representative lobules imaged to obtain mean scores for each experimental quarter sampled. Intralobular stroma and luminal invasion scores served as the dependent variables in two separate models using the MIXED procedure. Both models included the fixed, independent effects of quarter treatment (*n* = 2) and day euthanized (*n* = 2); the interactive term of quarter treatment and day euthanized was not included based on non-significance (*P* ≥ 0.30). Cow nested within day of euthanasia was specified as a random effect in both models, but group of cows (*n* = 3) was removed as a random effect because it was non-significant in all models when tested as a fixed effect (*P* ≥ 0.15). Resultant least squares means were contrasted using Fisher’s least significant differences test.

Measured tissue areas were used to calculate the percentage of lobule area occupied by: (1) intralobular stroma; (2) epithelial structures; and (3) luminal space for each lobule. These measures were subsequently averaged across the 8 representative lobules imaged to obtain mean percentages for each experimental quarter sampled. Intralobular stroma, epithelial structure, and luminal space percentages served as dependent variables in 3 separate models, which used the MIXED procedure. These models were identical to those previously described and used to analyze tissue immune cell invasion scores. Least squares means estimated by the models were contrasted using Fisher’s least significant differences test.

## Results

### Success of challenge

Intramammary *Staph. aureus* Novel challenge established IMI in 18 of the 19 infused quarters. All *Staph. aureus* infections persisted until tissues were collected at either 5 or 10 days post-challenge, and all saline infused quarters remained culture negative throughout. Challenged quarters did not display clinical signs of inflammation, such as quarters being red, swollen, or hot to the touch, but small flakes were occasionally observed in challenged quarter secretions. Secretion and tissue samples collected from the cow that did not develop an IMI in the challenged quarter were not utilized in any of the preceding described analyses. As a result, secretions and tissues were examined for 9 cows that were euthanized 5 days post-challenge and 9 cows euthanized 10 days post-challenge.

### Secretion somatic cells

**Figure 4:**
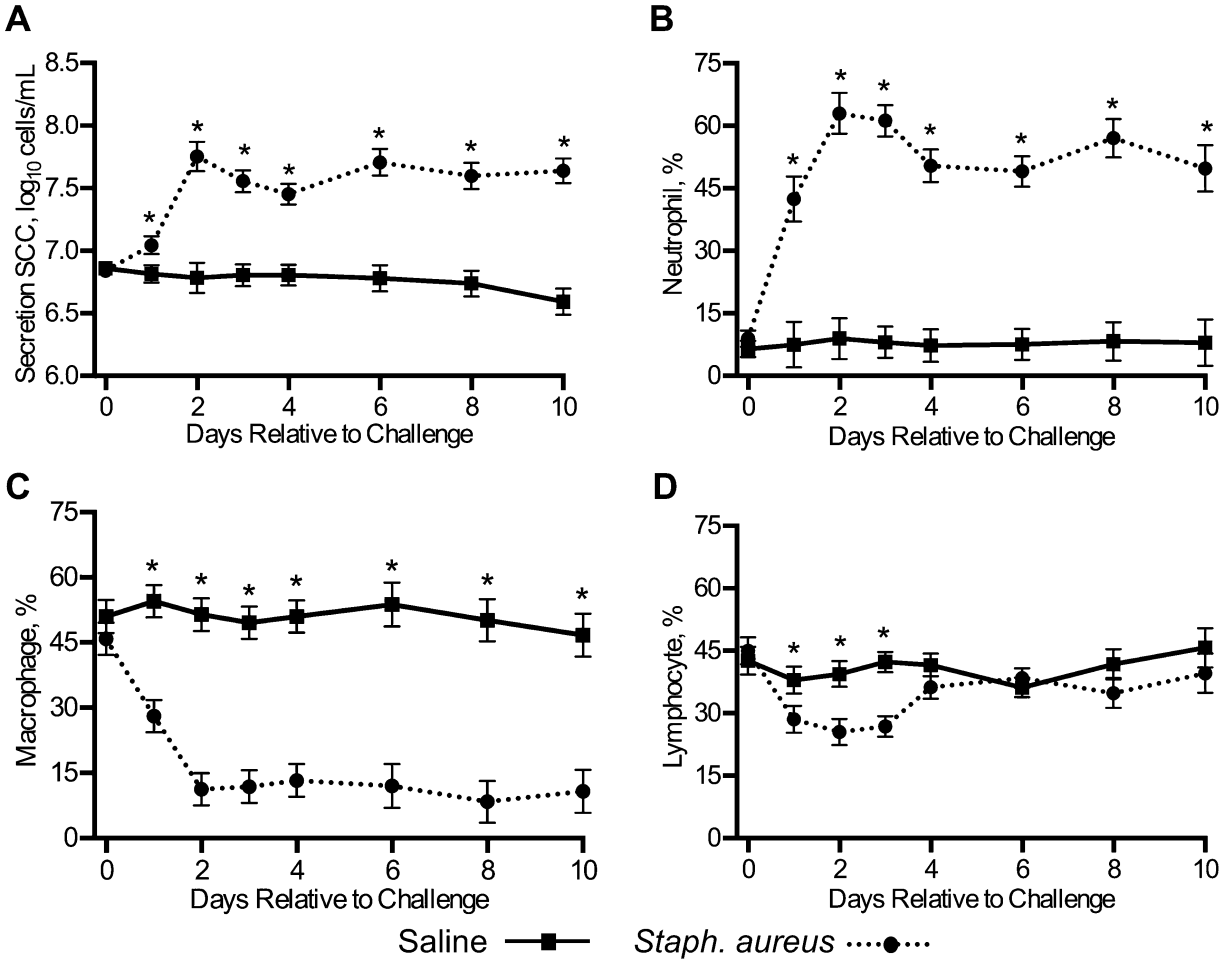
**Total SCC and differential cell type percentages in collected mammary secretions.** Total secretion SCC **A** and corresponding differential cell type percentages **B**–**D** collected from saline (*n* = 18) and *Staph. aureus* (*n* = 18) infused quarters. Error bars represent the standard error of the respective means. *P ≤ 0.05.

The mean secretion *Staph. aureus* quarter SCC (7.45 ± 0.06 log_10_ cells/mL) was greater than the mean saline quarter SCC (6.77 ± 0.06 log_10_ cells/mL; *P* < 0.001). Additionally, secretion SCC were significantly influenced by treatment interacting with trial day (*P* < 0.001; Figure 4A). Overall, secretion SCC appeared unchanged in saline quarters throughout the trial’s duration (Figure 4A) and these SCC were significantly lower for all days sampled post-challenge relative to challenged quarters (*P* < 0.05).

**Figure 5:**
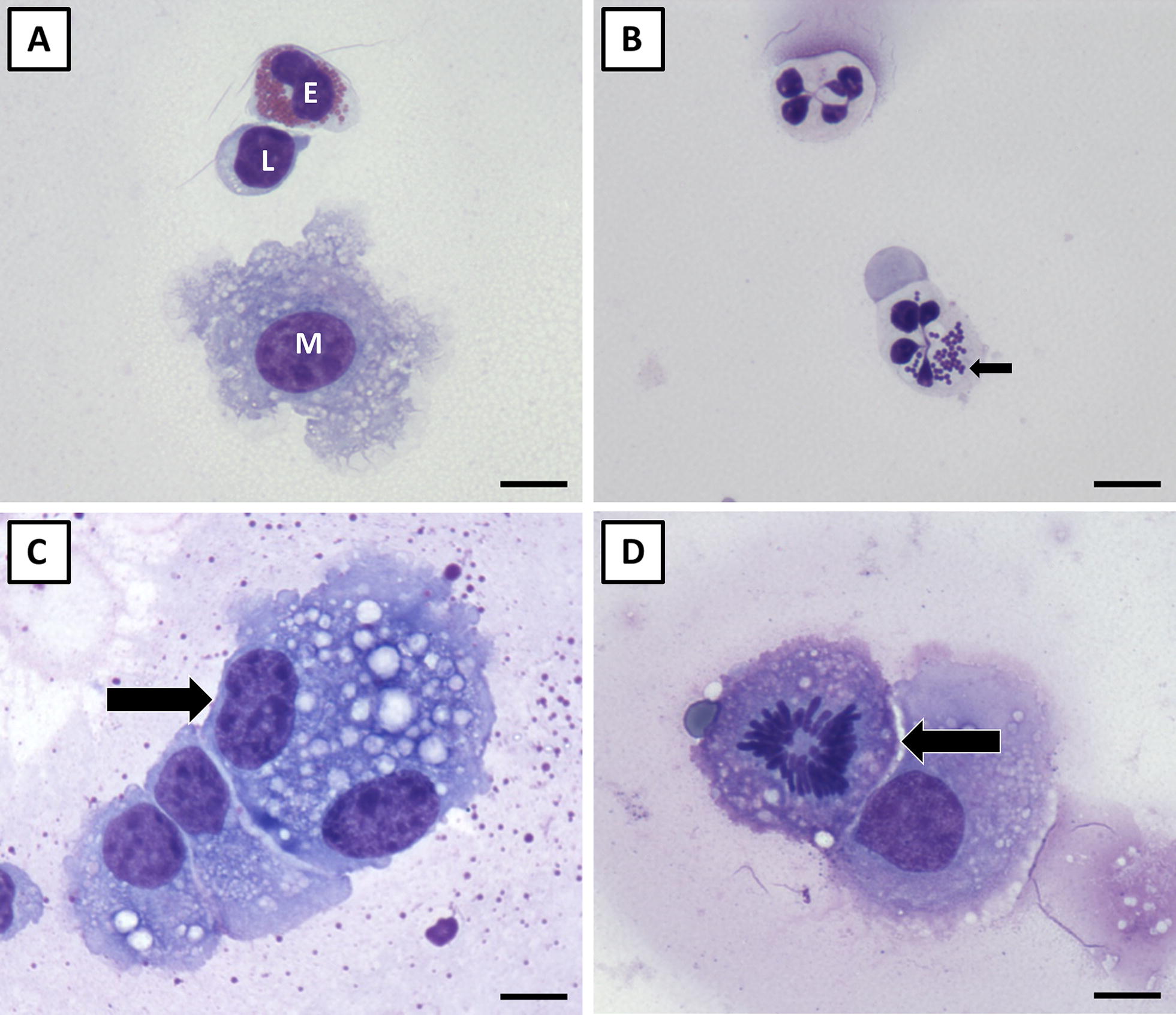
**Somatic cells observed in collected mammary secretions.** An eosinophil (E), lymphocyte (L), and macrophage (M) are depicted in **A**. **B** Depicts two neutrophils collected from a challenged quarter with the bottom neutrophil containing intracellular *Staph. aureus* (arrow). A binucleated macrophage is shown in **C** (arrow). These cells are suspected to originate from lumen resident macrophages that undergo incomplete cell division like the mitotic cell (arrow) shown in **D**. Scale bars = 10 µm.

Neutrophil, macrophage, and lymphocyte percentages measured in saline and challenged quarter mammary secretions are stratified by day of trial and are illustrated in panels B–D in Figure 4; representative images of each cell type are shown in Figures 5A and B. Overall, the mean percentage of neutrophils in challenged quarters (47.2 ± 2.3%) was greater than the mean neutrophil percentage in saline quarters (7.1 ± 2.3%; *P* < 0.001); conversely, the mean percentages of macrophages and lymphocytes in challenge quarters (17.7 ± 3.0% and 34.4 ± 2.1%, respectfully) were lower than those measured in saline quarters (51.0 ± 3.0% and 40.1 ± 2.1%, respectfully; *P* ≤ 0.03).

Aside from these main effects, a significant interaction existed between quarter treatment and trial day in its effect on all the measured cell type percentages (*P* < 0.001). In general, saline quarter cell type percentages remained stable throughout the trial, but cell type percentages in *Staph. aureus* quarters changed in response to challenge. For instance, neutrophil percentages were greater for every day sampled post-challenge in challenged quarters compared to saline quarters (*P* < 0.001; Figure 4B) and this appeared to impact macrophage percentages, which were lower in challenge quarters for every day sampled post-challenge (*P* < 0.001; Figure 4C). In addition, some neutrophils collected from challenge quarters in the present study were observed to contain intracellular *Staph. aureus* (Figure 5B). Lymphocyte percentages were lower in challenged quarters than saline quarters for the first 3 days following challenge (*P* < 0.05), but not for the remainder of the days sampled (*P* > 0.05; Figure 4D).

Eosinophils were also observed in secretions (Figure 5A) but made up less than 1% of the differential cell count, preventing comparisons from being made between saline and challenged quarters. Binucleated giant cells and lumen resident cells undergoing mitosis were also sporadically observed (Figures 5C and D).

### Tissue measures

**Figure 6:**
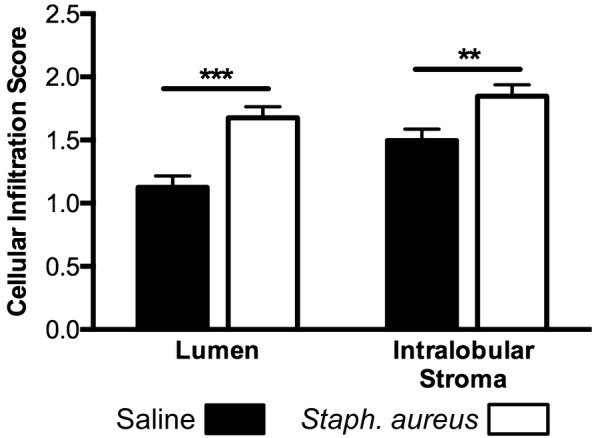
**Mean immune cell infiltration scores for lumen and intralobular stroma areas.** Mammary tissues were collected from 18 saline and 18 *Staph. aureus* infused quarters and 8 representative lobules were scored for each experimental quarter. Error bars represent the standard error of the respective mean immune cell infiltration scores. Asterisks denote differences between saline and *Staph. aureus* quarter treatments within intralobular stoma and luminal areas. **P ≤ 0.01, ***P ≤ 0.001.

**Figure 7:**
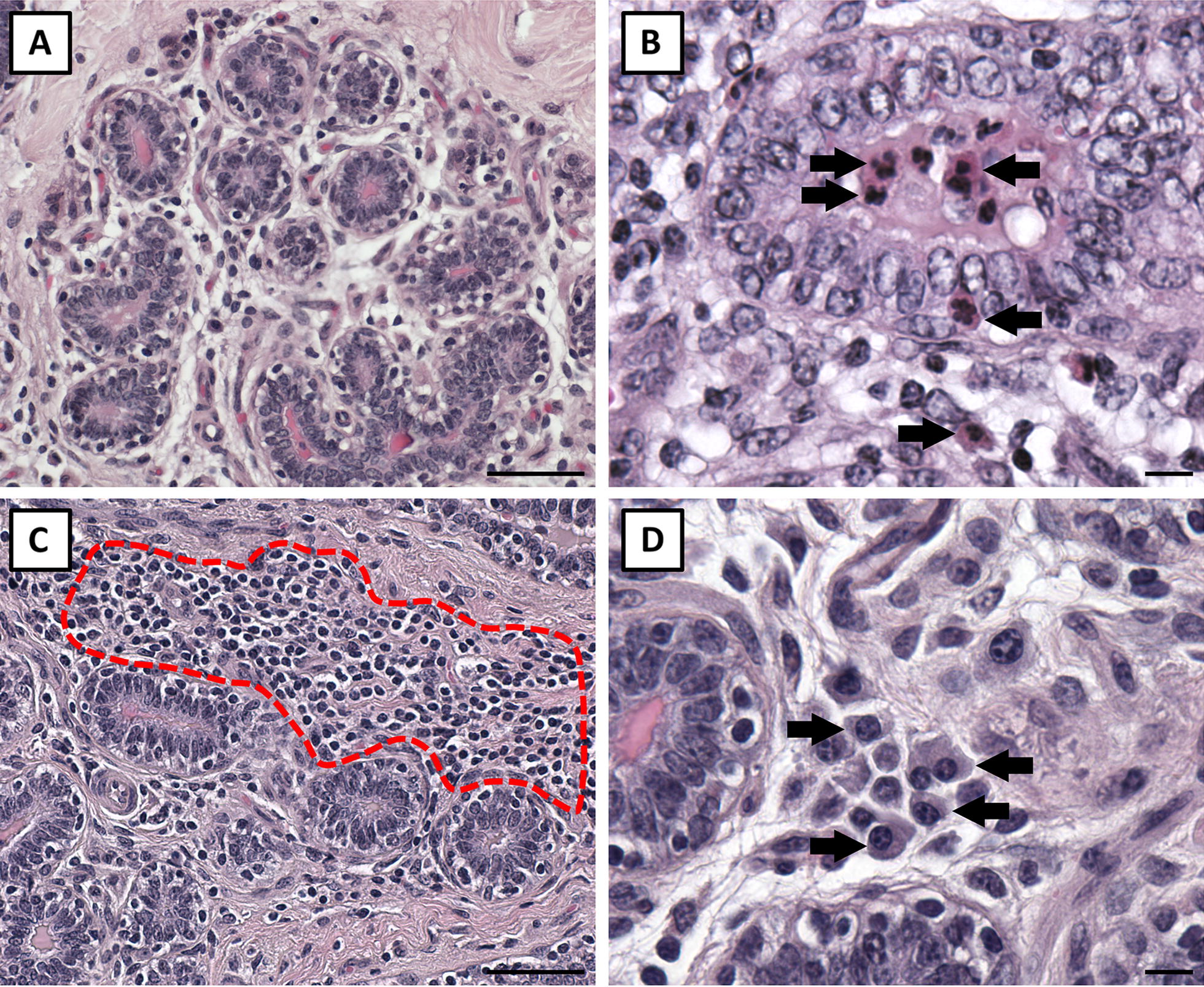
**Cellularity of tissues from saline and** ***Staph. aureus*** **quarter lobules.** **A** Depicts tissues from a saline infused quarter exhibiting diffuse intralobular stroma and non-secretory epithelium. Neutrophilic infiltration (arrows) of luminal and intralobular stoma compartments is depicted in **B** for tissues from a *Staph. aureus* infused quarter lobule. **C** Exemplifies the preferential infiltration of lymphocytes into intralobular stroma areas (dashed outline) that could be observed in saline quarters but were more frequent in *Staph. aureus* quarters. Plasma cells (arrows) could be observed in both saline and *Staph. aureus* quarter lobules **D**. Scale bars in **A** and **C** = 50 µm, **B** and **D** = 10 µm.

Immune cell infiltration scores were not affected by day of euthanasia (*P* ≥ 0.25) but were greater for challenged quarter lobules than saline quarter lobules for both the luminal (1.68 vs 1.13 ± 0.09; *P* < 0.001) and intralobular stroma compartments (1.85 vs 1.50 ± 0.09; *P* = 0.005) (Figure 6). Saline quarter lobules were essentially devoid of neutrophils in both the luminal and intralobular stromal compartments (Figure 7A), but neutrophils were frequently observed in both compartments of *Staph. aureus* challenged quarter lobules (Figure 7B). Lymphocytes could be observed in both saline and challenged quarters but were more abundant in the latter. It is noteworthy to state that lymphocytes appeared to preferentially accrue in intralobular stromal compartments rather than luminal spaces (Figure 7C). Plasma cells were abundant in both saline and challenged quarter tissues, but did not grossly appear to be more abundant in one vs the other (Figure 7D).

**Figure 8:**
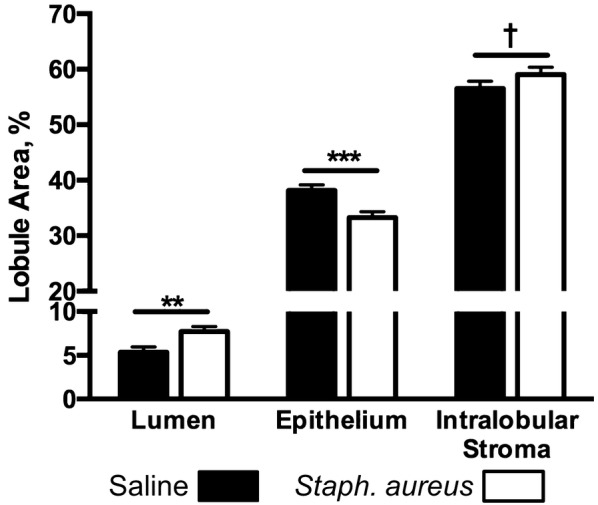
**Lobule area percentages occupied by luminal space, epithelium, and intralobular stroma in experimental quarters.** Mammary tissues were collected from 18 saline and 18 *Staph. aureus* infused quarters and 8 representative lobules from each quarter were used to quantify lobule areas occupied by intralobular stroma, epithelium, and luminal space. Error bars represent the standard error of the respective mean percentages. Dagger symbol and asterisks denote differences between saline *and Staph. aureus* quarter treatments within tissue structures; ^†^*P* = 0.1, **P ≤ 0.01, ***P ≤ 0.001.

Lobules in *Staph. aureus* challenged quarters exhibited a greater percentage of luminal space (7.7% vs 5.4% ± 0.6%; *P* = 0.004), a reduced percentage of epithelial area (33.3% vs 38.1% ± 1.1%; *P* < 0.0001), and tended to have a greater percentage of intralobular stromal area (59.0% vs 56.5% ± 1.3%; *P* = 0.1) than saline infused glands (Figure 8).

## Discussion

### Secretion somatic cells

The first objective of this study was to characterize the somatic cell and differential cell count response resulting from infusion of saline and *Staph. aureus* Novel into non-lactating mammary glands. The absence of an increase in secretion SCC in saline infused quarters was expected. This indicates that no significant immune response resulted from saline infusion and complements the observation that saline quarters remained culture negative throughout the trial. Conversely, the significant increase in secretion SCC observed in response to *Staph. aureus* challenge was expected and establishes that an immune response resulted in these quarters. No reports could be identified describing the SCC response of non-lactating mammary glands to *Staph. aureus* challenge but it has been reported that secretions collected from uninfected quarters of pregnant dry cows, contain a lower SCC than those collected from infected quarters [31]. Furthermore, the reported SCC of uninfected and infected quarters in the previous report [31] are comparable to the SCC observed here, indicating that the SCC response was similar to that of the pregnant dry cow. Aside from the SCC response in non-lactating glands, previous reports in lactating cows [32–34] have described increases in SCC resulting from *Staph. aureus* challenge.

The observed increase in neutrophil percentages in *Staph. aureus* infused quarters were expected given neutrophils are the main innate immune effector cell in the bovine mammary gland that respond to IMI [35] and similar changes have been described in other challenge trials in heifers [36] and lactating cows [34]. Neutrophils have also been described as being the predominate cell type in infected dry cow gland secretions [31]. Lymphocytes were the second most predominate cell type observed in challenged quarters but, this observation is not consistent with two previous reports [31, 37] that reported that macrophages were the second most predominant cell type infected dry cow secretions. Reasons for this disparity are unclear but are posited to be attributed to the fact that the secretions collected in those two previous studies were from quarters naturally and chronically infected with an assortment of different mastitis pathogens. The secretions collected herein were collected from quarters responding to a *Staph. aureus* challenge and are thus more associated with a rapid immune response rather than an immune response linked to chronic infections. Also, the immune response generated herein was specific to *Staph. aureus* and it has been previously demonstrated that different mastitis pathogens elicit different cytokine profiles and immune responses [38] which would, in part, explain the discrepancy between these results.

Binucleated giant cells, like those observed here, have been reported and discussed previously [26, 39, 40], but how these cells originate in the gland is not entirely clear. It is possible that a subset of these cells originated from what appear to be lumen resident macrophages undergoing incomplete cell division (Figure 5D) given that these mitotic cells were most commonly observed within secretions containing binucleated cells. Additionally, binucleated epithelial cells have also been described [41] and would contribute to the presence of these cells in milk or mammary secretions should they be sloughed from the basement membrane into the lumen.

### Tissue measures

The second key objective of this study was to characterize mammary tissue structure in quarters infused with saline and *Staph. aureus* to define how *Staph. aureus* challenge affected mammary gland structure and development. The observed infiltration of immune cells into *Staph. aureus* infected tissues was expected and complements the influx of immune cells into challenged quarter mammary secretions that would result from leukocyte diapedesis from blood vessels to mammary gland lumens in response to the presence of *Staph. aureus*. The abundance of plasma cells observed in mammary tissues from both treatments is believed to be consequence of the estradiol and progesterone injections given their significance in colostrogenesis [42, 43]. Not surprisingly, a similar hormonal induction model to that used here has been used to investigate bovine colostrogenesis mechanisms [44]. Examination of colostrum formation and immunoglobulin transport was not an objective in the present study but the abundance of plasma cells in the collected tissues may warrant consideration for future studies investigating colostrogenesis mechanisms, particularly those concerned with immunoglobulin production and transport.

No reports examining the histopathological response of a mastitis challenge in non-pregnant, dry cows could be identified with which to compare the tissue area percentages reported here. However, a previous report described the histopathological response of mammary tissue in non-pregnant heifers after *Staph. aureus* challenge [12] and reported a similar reduction in the epithelial areas relative to uninfected quarters. This previous study also reported that *Staph. aureus* infected tissues contained greater areas of stroma tissue than uninfected quarters and complements the tendency of *Staph. aureus* quarter lobules to contain greater areas of intralobular stroma than saline lobules as similarly reported here. Differing from the results of this previous study was the observation that challenged quarters exhibited larger luminal areas than saline infused quarters. The larger luminal areas observed herein are suspected to be consequence of the initial immune cell influx into the gland’s lumen, bringing fluid across the epithelium, given that IMI reduces epithelial integrity and results in increased concentrations of BSA [45] and ions [46] in milk from affected quarters. The reason for the lack of agreement between the previous study and the results of the present may also be attributed to differences infection duration. The cows used here were euthanized 5 and 10 days post-challenge, whereas the previous study euthanized heifers 2–3 weeks post-challenge [12]. This longer infection duration would have allowed the sustained immune response to affect glandular structure over a longer period of time. As a result, continued deposition and accumulation of connective tissues, leading to fibrosis, would result and begun to displace luminal space as fluid from infected quarters was reabsorbed, given the initial immune response had begun to subside.

The dry cows used in this study were treated with estradiol and progesterone to stimulate mammary growth and development so that IMI impact in growing and developing mammary glands could be investigated. In this context, the reductions in epithelial areas and tendency for challenged glands to contain greater areas of stromal tissue indicate that challenged glands failed to develop comparable amounts of epithelium and experienced varying degrees of connective tissue deposition in the gland as a result of IMI. It is currently unknown what chief mechanisms are responsible for these changes in glandular structure, but the deposition and accumulation of connective tissues in affected tissues, displacing mammary epithelium, and the immune response produced to address the presence of bacteria may interfere with mammary epithelial cell proliferation and alter gland development, perhaps in the long term. Such changes in glandular development are expected to contribute, in part, to the reduced milk yields reported for heifers that freshen with IMI [47, 48] as well as the reduced milk yields described for cow quarters that freshen with IMI compared to paired, uninfected, lateral quarters within the same cow [49].

Interestingly, neither day of euthanasia for tissue collection nor the interaction between day of euthanasia and treatment significantly influenced any of the respective lobule area percentages measured (*P* ≥ 0.18). This was unexpected, but is not entirely surprising given this study’s experimental design. This study was designed to first allow for treatment comparisons to be made within animal to control for inter-animal variation. When an examination of day of euthanasia was applied to this design, resulting in the nesting of animals within day euthanized, control for between animal variation was lost, which significantly influenced the studies ability to detect quarter treatment differences between animals euthanized at 5 or 10 days post-challenge. Furthermore, perhaps examining tissues 5 days post-challenge was too late to capture the temporal changes occurring in gland morphology resulting from intramammary *Staph. aureus* challenge; collection of tissues closer to the initial challenge may have allowed for changes over time in gland structure to be better appreciated.

In conclusion, *Staph. aureus* challenge of rapidly growing, non-lactating mammary glands increased immune cell invasion in mammary secretions and both intralobular stroma and luminal compartments of the mammary gland. This invasion was associated with changes in mammary structure as *Staph. aureus* challenged quarters exhibited reduced areas of epithelium and tended to have greater areas of intralobular stroma relative to saline infused quarters. When these histological changes are taken together, it suggests that IMI in rapidly growing non-lactating mammary glands limit mammary growth and development, which is expected to negatively impact future milk yield, milk quality, and productivity of the animal in the herd.

## Abbreviations

IMI: intramammary infection
SCC: somatic cell count
*Staph. aureus*: *Staphylococcus aureus*
PBS: phosphate buffered saline
BSA: bovine serum albumin
CFU: colony forming units
